# Neural Mechanisms Underlying the Cost of Task Switching: An ERP Study

**DOI:** 10.1371/journal.pone.0042233

**Published:** 2012-07-30

**Authors:** Ling Li, Meng Wang, Qian-Jing Zhao, Noa Fogelson

**Affiliations:** 1 Key Laboratory for NeuroInformation of Ministry of Education, School of Life Science and Technology, University of Electronic Science and Technology of China, Chengdu, China; 2 Department of Psychology, University of A Coruña, La Coruña, Spain; University of Granada, Spain

## Abstract

**Background:**

When switching from one task to a new one, reaction times are prolonged. This phenomenon is called ***switch cost (SC).*** Researchers have recently used several kinds of task-switching paradigms to uncover neural mechanisms underlying the SC. Task-set reconfiguration and passive dissipation of a previously relevant task-set have been reported to contribute to the cost of task switching.

**Methodology/Principal Findings:**

An unpredictable cued task-switching paradigm was used, during which subjects were instructed to switch between a color and an orientation discrimination task. Electroencephalography (EEG) and behavioral measures were recorded in 14 subjects. Response-stimulus interval (RSI) and cue-stimulus interval (CSI) were manipulated with short and long intervals, respectively. Switch trials delayed reaction times (RTs) and increased error rates compared with repeat trials. The SC of RTs was smaller in the long CSI condition. For cue-locked waveforms, switch trials generated a larger parietal positive event-related potential (ERP), and a larger slow parietal positivity compared with repeat trials in the short and long CSI condition. Neural SC of cue-related ERP positivity was smaller in the long RSI condition. For stimulus-locked waveforms, a larger switch-related central negative ERP component was observed, and the neural SC of the ERP negativity was smaller in the long CSI. Results of standardized low resolution electromagnetic tomography (sLORETA) for both ERP positivity and negativity showed that switch trials evoked larger activation than repeat trials in dorsolateral prefrontal cortex (DLPFC) and posterior parietal cortex (PPC).

**Conclusions/Significance:**

The results provide evidence that both RSI and CSI modulate the neural activities in the process of task-switching, but that these have a differential role during task-set reconfiguration and passive dissipation of a previously relevant task-set.

## Introduction

Executive control is an endogenous process necessary for performing various tasks in parallel, so that we are able to flexibly coordinate several processes at a time in order to accomplish a particular plan or goal [1]–[2]. In the past 40 years, researchers have explored the mechanisms underlying executive control in the field of cognitive science [1], experimental psychology [3], psychiatry, and neuropsychology [4]. The prefrontal cortex plays a key role in executive control by maintaining and processing information according to the requirements of the plan or goal at hand [1]–[2]. Task-switching evaluates the ability to adapt to the environment by updating the relevant task set, and switching to a new task. Hence, the task-switching paradigm has become a useful tool for evaluating executive control and its neural correlates [2], [5].

When switching from one task to another new task, reaction time becomes longer and error rates increase, a phenomenon known as switch cost [6]–[8]. Two theories have been proposed regarding the underlying mechanism of SC. First one is the task-set inertia (TSI) theory suggesting that the SC reflects a passive dissipation process from the previous task [7]. The second one is the task-set reconfiguration (TSR) theory suggesting that the SC is an active reconfiguration process for the new task [8]. Recently a new study challenged these traditional theories and reported that SC reflects an adaptation process to the abstract task representations by a parametric approach [9]. Thus, SC may reflect a mixed model of task dissipation and set. However, the neural mechanism remains largely undefined.

Task switch mainly include two processes: task-set reconfiguration and passive dissipation of the previously relevant task-set. Several paradigms have been used by researchers to study switch cost effects. The first behavioral study of task-switching was developed by Jersild [10], in which the performance in a single task block (consisting of only one task: AAAAAA) was compared with that in a mixed block (containing two tasks: ABABAB), showing that subjects have a generally worse performance during switching tasks. However, this paradigm confounded the effects of task-switching and working memory load. Allport and colleagues used a number and a stroop experiment to investigate task-switching [7], and found that increasing working memory load did not significantly affect the switch cost. This revealed that an exogenous, not an executive control process, plays a key role in task-switching. In contrast, studies using an alternating-runs paradigm (AABBAABB) suggested that switch cost reflects the control of both exogenous and endogenous components [8], [11]. In turn, Merian and colleagues used a cued task-switching paradigm [12], with an unpredictable task switch, which dissociated task set, determined by CSI, and dissipation processes, determined by response-cue interval (RCI). By manipulating the CSI and RCI, decreases in switch cost were observed by increasing the CSI while keeping a constant response-stimulus interval (RSI), increasing the CSI while keeping a constant RCI, or increasing the RCI while keeping a constant CSI. Thus, multiple processes have been implicated to be involved in the switch cost.

A number of electrophysiological task-switching studies have been commonly used to explore different aspects of cognitive control [5], and other phenomenon such as preparation effect and residual cost. [13]. RCI and CSI are important for determining the size of switch cost in a cued task-switching paradigm [12], [14]. Though several sub-components underlying the switching process are still under dispute, studies using event-related potentials (ERP) provide neural correlated information for further understanding SC mechanisms [14]–[16]. One dominant ERP component in the cued task-switching task is a positive potential that is time-locked to the onset cue and is larger for switch relative to repeat trials [14]–[20]. This cue-locked switch-positivity appears about 300 ms after cue onset and peaks at posterior areas, suggested to reflect processes of anticipatory task-set reconfiguration [14]–[16]. Another ERP component is a stimulus-locked switch-negativity derived by subtracting repeat from switch trials. Stimulus-locked switch-negativity is related to post-stimulus processes initiated by the stimulus itself, and to some degree, the onset is also determined by the length of the preparation interval. Both ERP components differ in scalp distribution, onset, and duration, depending on the experimental paradigm [14]–[23]. Above ERP studies utilized unpredictable tasks with balanced working memory load, for example, switching between letter and digit tasks by spatial cues [12], [14], [15]. However, these paradigms were not able to dissociate the effects of task-switching and spatial attention. Other studies introduced confounding effects of task-switching and long-term memory by using color as a cue to switch between parity and magnitude [19].

Many neuroimaging studies have suggested that the dorsolateral prefrontal cortex (DLPFC), the anterior cingulated cortex (ACC), and the posterior parietal cortex (PPC) play an important role in subserving task-switching [24]–[30]. DLPFC has been associated with top-down processes related to the demand of control resources and representing and maintaining a task-set, while ACC is responsible for conflict-monitoring [24]. According to the TSR theory, an increase in the preparation-related activation of PFC, PPC, and ACC was observed for switch trials compared with repeat trials. Another study suggested that the task-switching process may consist of a switch-relevant transient control related to left lateral PFC and superior parietal cortex, and a mixing status-sensitive sustained control related to right anterior PFC [5]. Other findings suggested that task-set reconfiguration may consist of goal-activation and rule-activation processes related to DLPFC and PPC, respectively [31]. Thus, DLPFC has been shown to have a key role in processing of task-relevant stimuli, stimulus-response mappings and inhibition of irrelevant information [29], [32], while, PPC is responsible for adjusting a stimulus-triggered task-set [25], reconfiguring stimulus-response mappings [33] and transforming multi-modal inputs to a detailed motion plan [30].

The purpose of the current study was to explore the role of RSI and CSI on the sub-processes of task-set reconfiguration and passive dissipation of the previously relevant task-set. We hypothesized that, (a) RSI would play a key role in modulating the neural activities in the process of passive dissipation, and CSI would play a key role in modulating the neural activities in the process of task-set reconfiguration, (b) parietal and frontal cortices would be engaged in task-switching. To this end, we modified the task-switching paradigm, modulated RSI and CSI, analyzed cue-related and stimulus-related ERP waveforms, and utilized the standardized low resolution brain electromagnetic tomography (sLORETA) technology [34] to explore the role of RSI and CSI, and the role of the cortical areas involved in task-switching.

## Materials and Methods

### Subjects

Fourteen right-handed subjects (males) participated in the present study for monetary compensation. The mean age was 25 years, ranged between 20 and 27 years. The subjects had normal or corrected-to-normal color vision and no history of neurological problems. This study was reviewed and approved by the Institutional Review Board of UESTC (the University of Electronic Science and Technology of China) and an informed consent was signed and obtained from each subject before the experiment.

### Stimuli and Task

**Figure 1:**
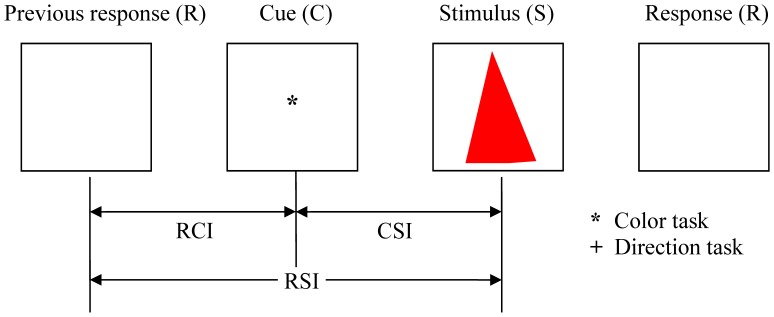
Schematic experimental paradigm. Each trial began with a cue and ended with the response. Once a response to a previous trial is completed, the cue for a current trial is presented with a designated duration, and it is then followed by a colored and oriented triangle stimulus. Cue-stimulus interval (CSI), response-cue interval (RCI), and response-stimulus interval (RSI) were defined as the interval between current cue and stimulus, previous response and cue, previous response and stimulus, respectively.

The stimuli were triangles combined by two types of color (red and green) and two types of direction (up and down). Subjects were instructed to switch between the color and orientation discrimination task using sign cues. To avoid the possibility of subjects using a memorization strategy to perform the task, we introduced a large range of stimulus color and orientation, to encourage the use of general rules rather than ‘check tables’ for solving the tasks. The color type ‘red’ included six similar colors (red, orange, pink, brown, rose, and salmon pink), and the color type ‘green’ included six similar colors (green, sea green, emerald green, aqua, turquoise, and cyan). There were 21 directions facing up and 21 directions facing down (orienting vertically up or down within ±10 degree). Targets contained four types of triangles: red up (RU), red down (RD), green up (GU), and green down (GD), which were presented with equal probability. Figure 1 shows a schematic experimental paradigm. Trials began with a central cue (*or +), after which subjects were presented with a randomly selected target, a colored and oriented triangle. A color detect task was cued by ‘*’; while ‘+’ indicated a discrimination of the orientation of the target stimulus. RCI, CSI and RSI were defined as the interval between a previous response and a cue, between a cue and a stimulus, and between a previous response and a stimulus, respectively.

**Figure 2:**
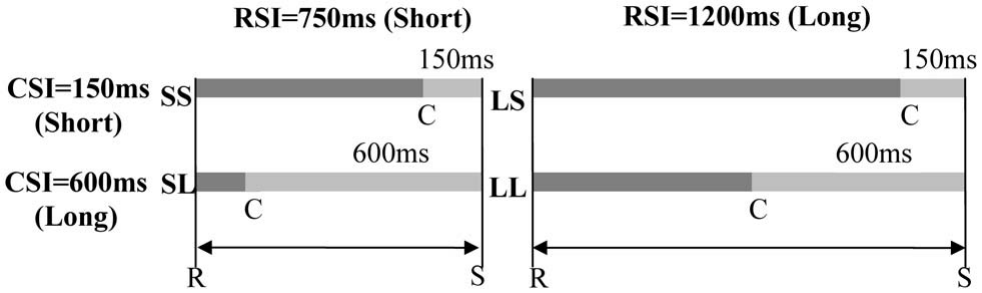
The parameters of RCI, CSI and RSI. Two levels of RSI and CSI were combined to produce 4 sessions: short RSI and short CSI (SS), short RSI and long CSI (SL), long RSI and short CSI (LS), and long RSI and long CSI (LL). In these four conditions, RCI included short (150 ms), long (600 ms), and longer (1050 ms) intervals.

Figure 2 illustrates the parameters of CSI and RSI. The two levels of RSI (long for 1200 ms; short for 750 ms) and CSI (long for 600 ms; short for 150 ms) were combined to produce the following 4 sessions: short RSI and short CSI (SS); short RSI and long CSI (SL); long RSI and short CSI (LS); long RSI and long CSI (LL). For each block, CSI and RSI were held constant, and a Latin square design was used to avoid sequence effects.

**Figure 3:**
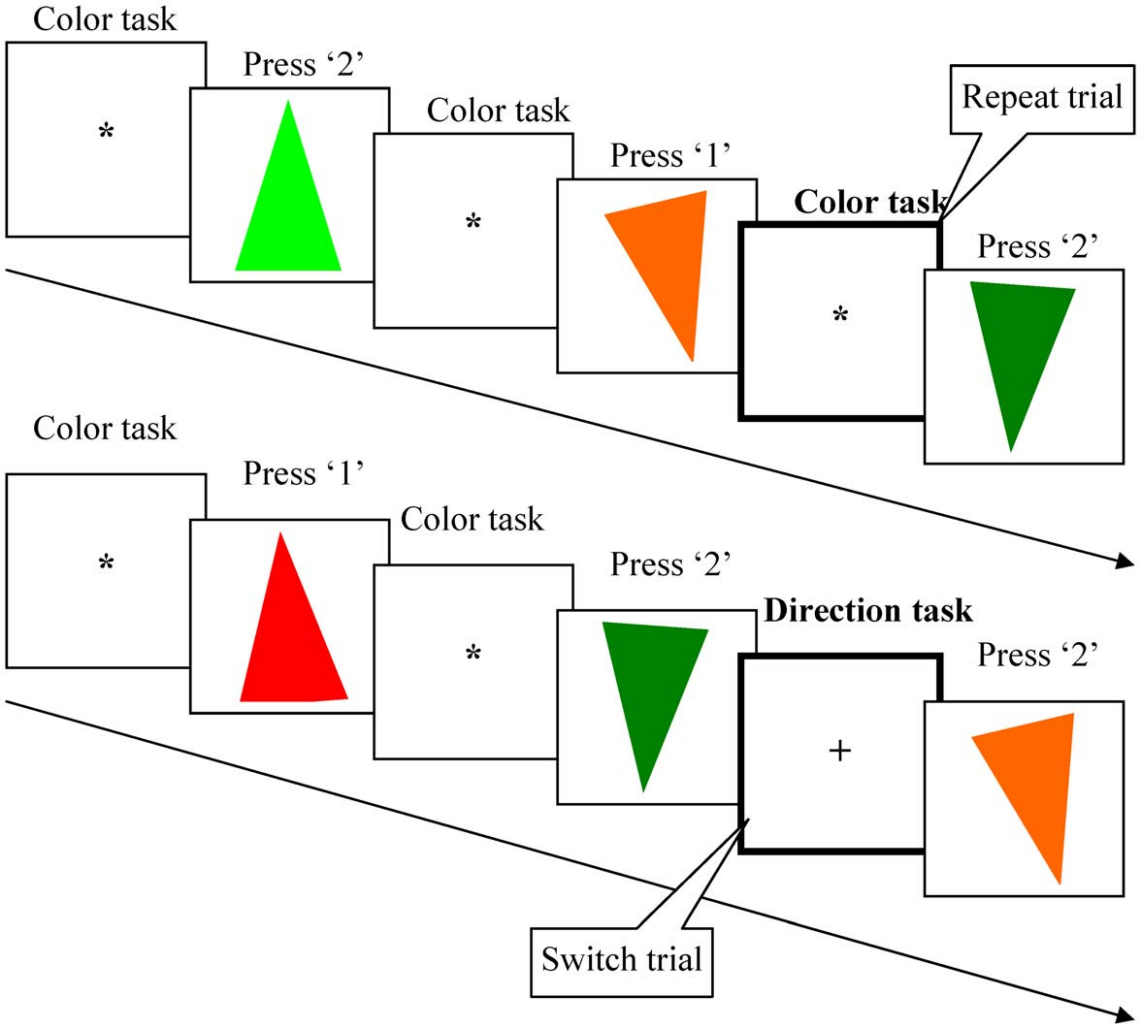
Examples of repeat and switch trials. Repeat trials were defined when a cue in the current trial was the same as the one in the previous trial, indicating that the same task should be performed (top row in the figure). Switch trials were defined when a cue in the current trial was different from the previous trial, indicating that the task should be switched (bottom row in the figure).

Figure 3 shows an example of the repeat and switch trial sequences. In both trial sequences, the cue was presented in the center of the screen, followed by a stimulus disappearing immediately upon a response, and then the cue in the next trial was presented after a designated time had elapsed. The same stimulus never repeated on the successive trials. When a previous cue was the same as the current cue, it was defined as a repeated trial. When a previous cue was different from the current cue, it was defined as a switch trial. The right index finger and middle finger were used to respond to button 1 and button 2, respectively. In the color task subjects pressed button 1 for red and button 2 for green (button 2), and in the orientation task subjects pressed button 1 for triangles pointing up and button 2 for triangles pointing down. Thus, half of the stimuli were congruent (mapped to the same finger in either task), no matter what task was relevant (red triangles pointing up, green triangles pointing down), while in the other half, different fingers were used to respond correctly to the cued tasks (green triangles pointing up, red triangles pointing down).

### Procedure

All subjects were seated comfortably, 80 cm away from a 21-inch video monitor, and were required to focus on the center of the screen in an acoustically and electrically shielded room. During the training session, all subjects started with two single-task blocks. Half of the participants first performed the color task block, while the other half began with the orientation task block. The subjects then performed the mixed-task blocks comprising of 80 trials with 8 switch trials each block. The subjects began with one task and switched to the other task after a minimum of 6 and maximum of 12 intervening trials. Half of the subjects started with the color task in the mixed block, while the other subjects began with the orientation task. Subjects were instructed to react as fast and accurately as possible and were given enough practice to achieve a level of 90% accuracy to ensure a familiarity with the cue-task and the response-finger mapping. After this 4 formal sessions which consisted of 8 mixed blocks were conducted in the same order as the training session. The first tasks in the successive blocks were alternated, for example, the first block began with the color task, and the next block started with the orientation task. Overall 4 sessions were included in the formal experiment, in which 576 repeat trials and 64 switch trials were recorded in each condition. During the experiment, subjects had to pay attention to the center of the screen and to identify the cue in order to respond accurately, so focal attention was required. The cue-task, response-finger and finger-key mappings were held constant for all the participants.

### EEG Recording and Data Preprocessing

Behavioral and continuous EEG were recorded using EGI system with a 128 channel electrode cap. Sampling rate was 1000 Hz, and band-pass was 0.1∼48 Hz. All scalp channels were referenced to Cz (129^th^) electrode. Trials were rejected if they had an incorrect response or lacked a button press between 300 and 1500 ms after the onset of the stimulus. Eye movements, blinks and muscle artifact were excluded by automatic artifact rejection (greater than 100 µV). After this preprocessing, a mean of 431±32, 408±31, 412±27, and 423±36 repeat trials remained across subjects for SS, SL, LS and LL conditions, respectively (mean ±SD). For switch trials, a mean of 52±6, 52±4, 51±6, and 54±7 trials remained across subjects for SS (range from 38 to 64), SL (range from 45 to 60), LS (range from 45 to 61) and LL (range from 44 to 64) conditions, respectively (mean ±SD). There was no significant difference between the number of switch trials across the four conditions (paired t-test, *p*>0.05).

Cue-related and stimulus-related waveforms were extracted by segmenting from 200 ms before the onset of the cue to 800 ms after the onset of the cue and from 200 ms before the onset of the stimulus to 800 ms after the onset of the stimulus, respectively. Baseline was set to 200-ms pre-onset of the cue and stimulus. All the data was transformed to average reference. Individual averaged ERPs for the cue and stimulus were acquired by selectively averaging across both tasks (color and direction) for each of the 4 conditions (SS, SL, LS and LL) in repeat and switch trials. Hence there were 8 cue-related and 8 stimulus-related average ERP waveforms for each subject. Difference waveforms for each participant were derived by subtracting the grand average waveforms for repeat trials from the grand average switch waveforms, so that 4 cue-related and 4 stimulus-related difference waveforms for each of the four conditions were obtained.

### Behavioral Data Analysis

The first trial of every block was excluded prior to any further analysis. Trials in which responses were inaccurate were defined as errors, and the trials following errors were also excluded from the RT analysis. RT was only measured for correct responses. Error rate was calculated to investigate whether there was a trade-off effect between RT and error rate. Switch cost of RTs was calculated by subtracting RT measured in previous repeat trials from current switch trials. Switch cost of error rates was evaluated by subtracting the error rate measured in previous repeat trials from current switch trials.

The relative contribution of the processes underlying CSI or RSI to switch cost (switch costs of RTs and error rates) was evaluated using a 2 CSI (short: 150 ms, long: 600 ms) ×2 RSI (short: 750 ms, long: 1200 ms) repeated measure ANOVA. Post-hoc parametric paired t-tests were performed with Sidak correction for multiple comparisons. To test the TSR [8] switch cost of RTs in the short and long CSI conditions were compared to examine whether it decreased in the long CSI. To test the TSI theory [7] and examine whether switch cost decreased in the long RSI, we compared switch cost in the short and long RSI conditions.

### ERP Analysis

The cue-related ERP at midline sites (Fz, Cz, Pz) was analyzed for switch and repeat trials in each of the 4 conditions. The mean amplitudes of the switch-related positivities recorded at Pz were extracted for further analysis. A 2 trial type (repeat, switch) ×2 CSI (short: 150 ms, long: 600 ms) ×2 RSI (short: 750 ms, long: 1200 ms) repeated measure ANOVA was used to compare ERP amplitudes and post-hoc parametric paired t-tests with Sidak correction were used for multiple comparisons. The mean differential amplitudes between switch and repeat trials for each condition (switch cost of ERP amplitudes) were calculated to examine the differential effect of RSI and CSI.

The stimulus-related ERP and difference waveforms at midline sites (Fz, Cz, Pz) for repeat and switch trials were analyzed in the different CSI conditions. The mean amplitudes of the switch-related negativities at Cz (or Pz) were evaluated and compared across different conditions using a 2 trial type (repeat, switch) ×2 CSI (short: 150 ms, long: 600 ms) ×2 RSI (short: 750 ms, long: 1200 ms) repeated measure ANOVA. Post-hoc parametric paired t-tests were performed with Sidak correction for multiple comparisons. Switch cost of ERP amplitudes was also calculated to examine the differential effects of RSI and CSI. Mean values ± mean standard error of the mean (SEM) are used throughout the text. Pearson correlation was performed to test the relationship between the SC of reaction times, SC of cue-related amplitudes, and SC of stimulus-related amplitudes in the four conditions.

### sLORETA Analysis

sLORETA algorithm solves the inverse problem using scalp electric potential (EEG) to find the “smoothest” possible 3D distribution of the neural electric activities. We applied the sLORETA software to perform voxel-by-voxel between-group comparisons of the switch-related positivities and negativities. A total of 6430 voxels (at each voxel, three dipole moments were estimated) at 5 mm spatial resolution were generated. Specifically, cue-related and stimulus-related brain activities between switch and repeat trials were compared to examine the areas involved in the switching process in each condition. Brain activities in repeat and switch trials between short and long CSI conditions were compared to examine the regions associated with the CSI. Nonparametric statistical analyses (Statistical non-Parametric Mapping, SnPM) were used employing a t-statistic for paired groups (repeat and switch). The results of SnPM are described as t-values for each voxel with Bonferroni correction for multiple comparisons. Corrected *p*<0.05 values were accepted as statistically significant.

## Results

### Behavioral Results

**Figure 4:**
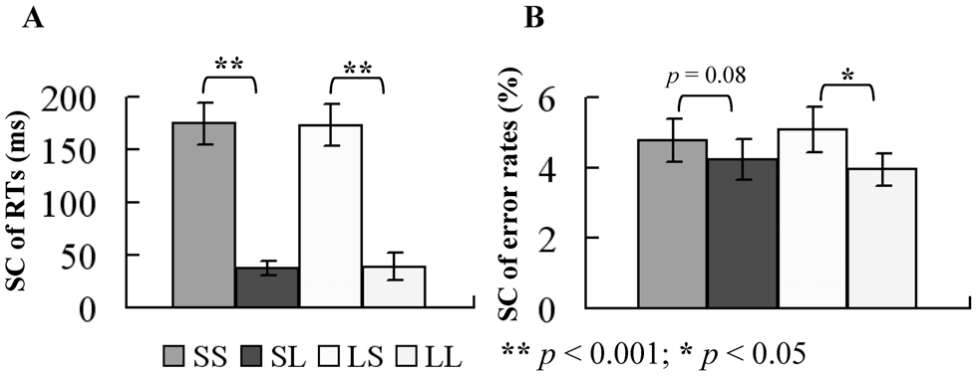
Behavioral switch cost. (A) Average switch costs of behavioral reaction time are shown for the 14 subjects in the four conditions. Switch cost was smaller in the long CSI condition. (B) Average switch costs of behavioral error rates are shown for the 14 subjects in the four conditions. Subjects responded more accurately in the SL and LL conditions (long CSI condition). Switch cost was smaller in the long CSI. Error bars show SEM.

The ANOVA revealed a main effect of CSI condition for SC of RTs, in which long CSI resulted in a significant decrease in SC of RTs (*p*<0.001), but no such effect was present for RSI condition. The switch cost of RTs between previous repeat trial and current switch trial was 174.58±19.60 ms, 37.33±7.21 ms, 173.18±20.01 ms, and 39.05±13.01 ms for SS, SL, LS, and LL conditions, respectively (mean ± standard error of mean (SEM) for this and all following results). There was a main effect of CSI condition for SC of error rates (*p*<0.05) with lower SC overall in the long CSI condition, but no such effect was present for RSI condition. Switch cost of error rates between previous repeat trial and current switch trial was 4.78±0.62%, 4.22±0.58%, 5.08±0.66%, and 3.94±0.45% for SS, SL, LS, and LL conditions, respectively. Long CSI resulted in a marginal significant decrease in switch cost of error rates (*p = *0.06). There was no interaction between CSI and RSI (*p*>0.05) for switch cost of RTs and error rates. Switch cost of RTs and error rates for each condition are illustrated in Figure 4A–B.

### ERP Results

#### Cue-related ERP

**Figure 5:**
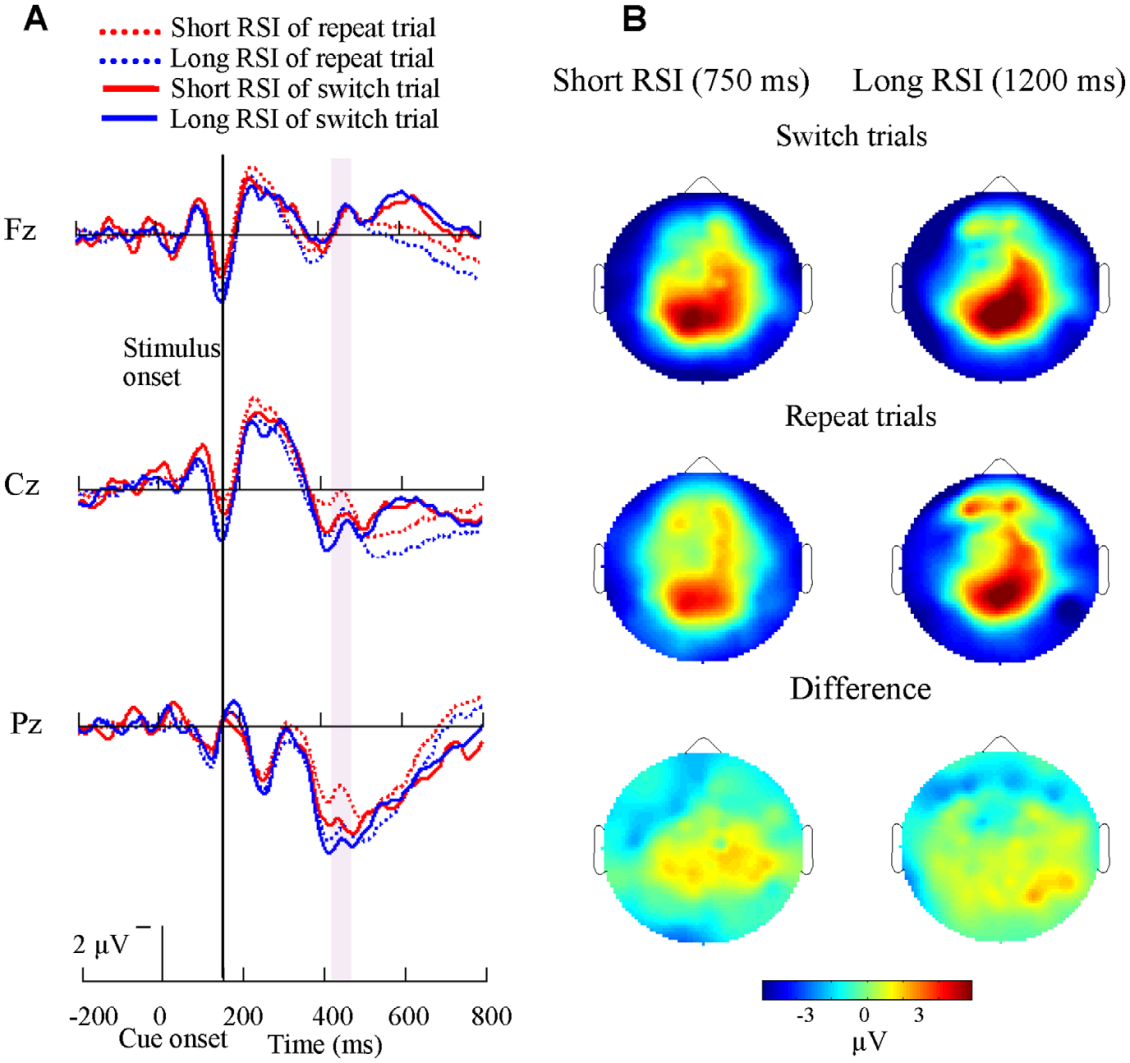
Cue-related ERP waveforms and brain topographies for short CSI conditions. (A) Grand-average cue-related ERP waveforms at midline sites (frontal, Fz, central, Cz, and parietal, Pz) are shown for the 14 subjects in the short CSI conditions across both repeat and switch trials. A large sharp parietal positivity emerged at about 400 ms after cue onset. Switch trials evoked larger amplitude than that of repeat trials. (B) Brain topographies in the short CSI conditions across repeat (mid) and switch trials (top) and the difference topographies between switch and repeat trials (bottom) are shown. There was more activation for switch trials in parietal cortex.

**Figure 6:**
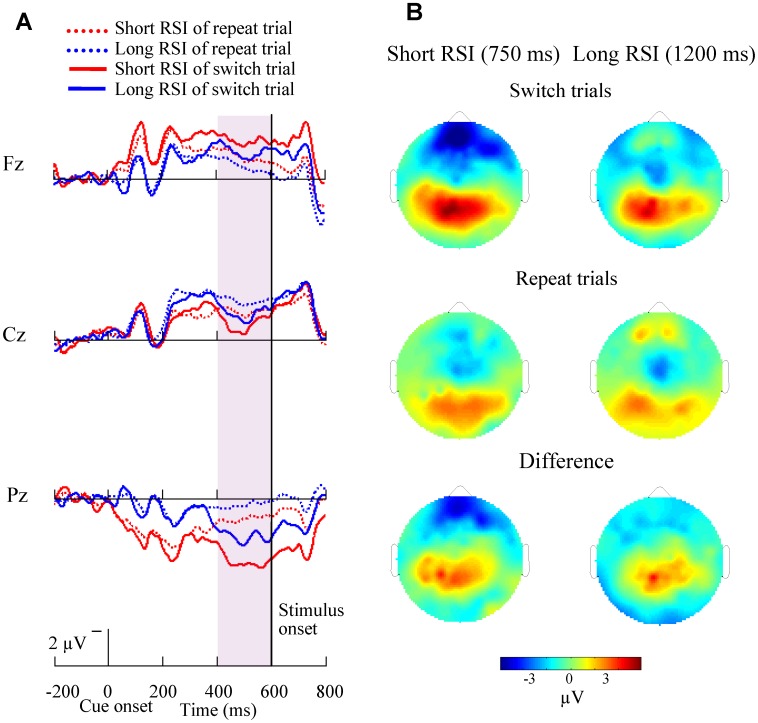
Cue-related ERP waveforms and brain topographies for long CSI conditions. (A) Grand-average cue-related ERP waveforms at midline sites (Fz, Cz, Pz) are shown for the 14 subjects in the long CSI conditions across both repeat and switch trials. A slow parietal positivity emerged at about 400 ms after cue onset. Switch trials evoked larger amplitudes than that of repeat trials. (B) Brain topographies in the long CSI conditions across repeat (mid) and switch trials (top) and the difference topographies between switch and repeat trials (bottom) are shown. There was more activation for switch trials in parietal cortex.

The grand-average ERPs time-locked to cue onset at midline sites (Fz, Cz, Pz) and the topography of mean amplitude (425–475 ms time-window) of the switch-related positivities across all 14 subjects are illustrated in Figure 5A–B for switch and repeat trials in the short CSI (150 ms) condition. A parietal maximal sharp positivity emerged and peaked around 450 ms after cue onset. Figure 6A–B illustrats cue-related ERP at Fz, Cz, Pz electrodes and the topography of mean amplitude (400–600 ms time-window) of the switch-related positivities for both types of trials in the long CSI (600 ms) condition. A slow positivity was induced 400–600 ms after cue onset for the long CSI condition, for both short and long RSI.

The mean amplitudes of the switch-related positivities for both CSI conditions recorded at Pz were evaluated (Table S1). There was a main effect of trial type and CSI condition with smaller amplitudes in the long CSI condition (*p = *0.001) and the repeat trial (*p*<0.001), but no such effect was present for RSI condition. Interaction between RSI and trial type was significant (*p = *0.045), showing an increased decline in amplitude for the repeat trial compared with the switch trial in the short RSI condition compared with the long RSI condition. A switch-related positivity was observed indicated by larger amplitudes evoked by switch trials compared with repeat trials at parietal sites.

**Figure 7:**
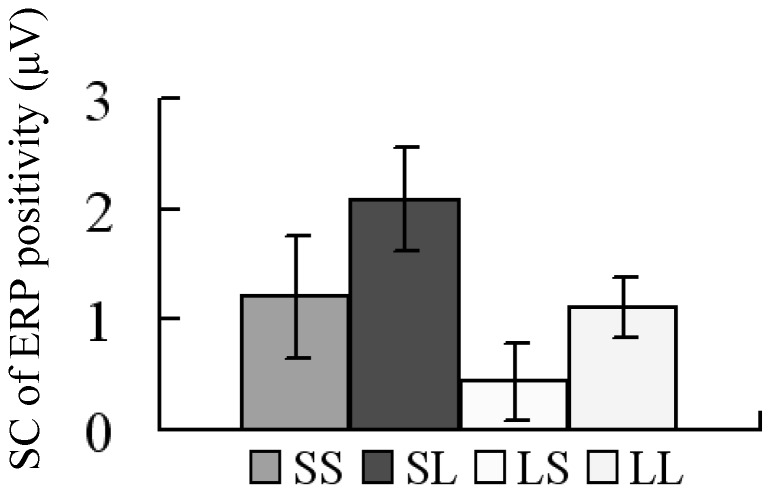
Switch cost of cue-related ERP positivity. Switch cost of ERP positivity was evaluated by subtracting amplitude of the switch-related positivity for repeat trials from that for switch trials. Long RSI resulted in a smaller SC of ERP positivity.

The ANOVA comparing SC of ERP positivity revealed a main effect of RSI condition, in which long RSI resulted in a smaller SC of ERP positivity (*p = *0.045) compared with short RSI, but no such effect was observed for the CSI condition (*p = *0.075). Interactions between CSI and RSI were not significant (*p*>0.05). Switch cost of ERP amplitudes were 1.21±0.55 µV, 2.08±0.47 µV, 0.44±0.35 µV, and 1.11±0.27 µV for SS, SL, LS, and LL conditions, respectively (illustrated in Figure 7).

#### Stimulus-related ERP

**Figure 8:**
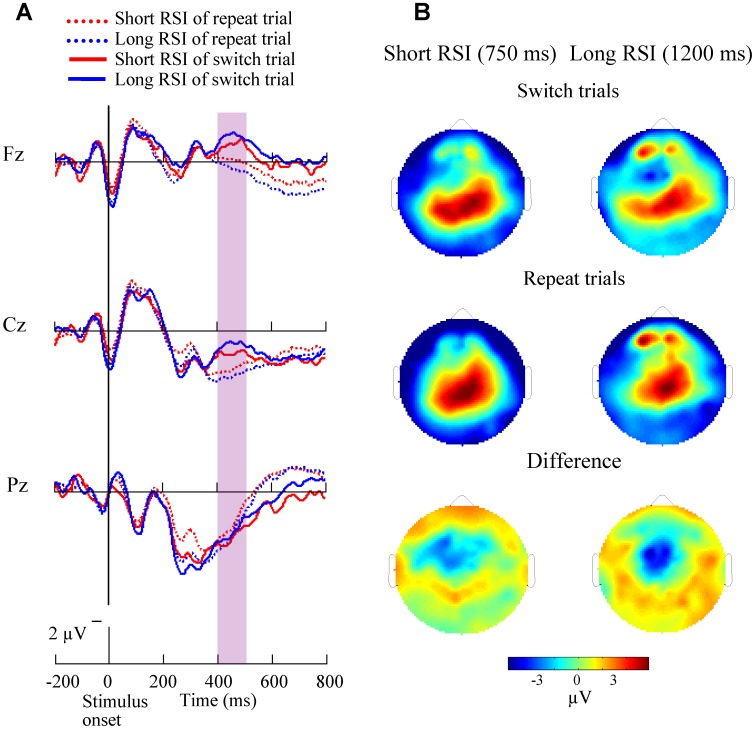
Stimulus-related ERP waveforms and brain topographies on short CSI condition. (A) Grand-average stimulus-related ERP waveforms at midline sites (Fz, Cz, Pz) are shown for the 14 subjects in short CSI conditions across both repeat and switch trials. In all the repeat and switch trials, a large sharp central and frontal negativity emerged at about 400 ms after stimulus onset. (B) Brain topographies in the short CSI conditions across repeat (mid) and switch trials (top) and the difference topographies between switch and repeat trials (bottom) are shown.

**Figure 9:**
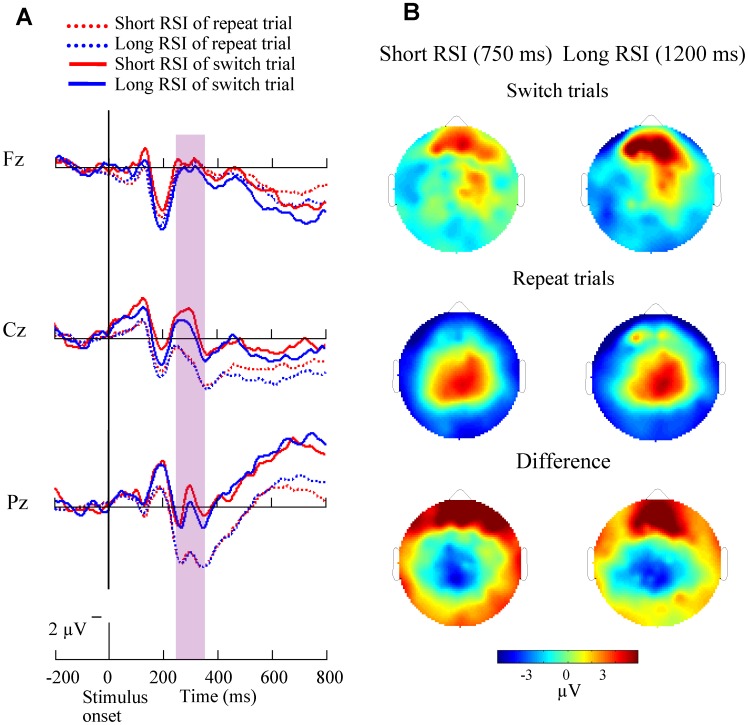
Stimulus-related ERP waveforms and brain topographies on long CSI condition. (A) Grand-average stimulus-related ERP waveforms at midline sites (Fz, Cz, Pz) are shown for the 14 subjects in long CSI conditions across both repeat and switch trials. In all the repeat and switch trials, a slow central and parietal negativity emerged at about 300 ms after stimulus onset. (B) Brain topographies in the long CSI conditions across repeat (mid) and switch trials (top) and the difference topographies between switch and repeat trials (bottom) are shown.

The grand-average ERPs time-locked to stimulus at midline sites (Fz, Cz, Pz) and the topography of mean amplitude (400–500 ms time-window) of the switch-related negativities across all 14 subjects are displayed in Figure 8A–B for switch and repeat trials in the short CSI (150 ms) condition. Both repeat and switch ERP waveforms showed a sharp large positivity which emerged around 275–325 ms after stimulus onset at central and posterior parietal sites. The switch-related negativity was induced 400 ms after stimulus onset at the frontal and central areas. Figure 9A–B illustrates stimulus-related ERP at Fz, Cz, Pz and the topography of the mean amplitude (250–350 ms time-window) of the switch-related negativities for both types of trials in the long CSI (600 ms) condition. A parietal maximal sharp switch-related negativity emerged and peaked around 300 ms after stimulus onset.

The absolute mean amplitudes of the switch-related negativities for short CSI recorded at Cz and for long CSI recorded at Pz site were evaluated (Table S2). There was a main effect of trial type and CSI condition, with smaller amplitudes in the short CSI condition and in the switch trial (*p*<0.001) compared with long CSI and repeat trial, respectively. However, there was no significant effect for the RSI condition. Interaction between CSI and trial type was significant (*p = *0.003), showing an increased decline in amplitude for the switch trial compared with the repeat trial in the long CSI condition compared with the short CSI condition. Repeat trials evoked larger amplitudes than that of switch trials in the long CSI condition (*p*<0.05), indicating a switch-related negativity.

**Figure 10:**
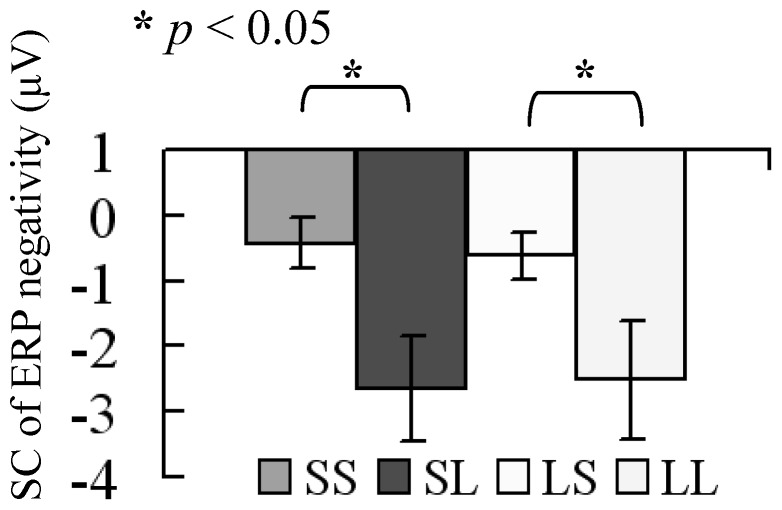
Switch cost of stimulus-related ERP negativity. Switch cost of ERP negativity was evaluated by subtracting the amplitude of the switch-related negativity for repeat trials from that for switch trials across the four conditions (SS, SL, LS, and LL). CSI had main effects for SC. SC of neural activity was smaller in the long CSI condition.

The ANOVA revealed a main effect of CSI condition for SC of ERP negativity, in which long CSI resulted in a significant smaller SC of ERP negativity (*p = *0.003), but no such effect was present for RSI condition (*p = *0.964). Interactions between CSI and RSI were not significant (*p*>0.05). Switch cost of ERP amplitudes was −0.43±0.39 µV, −2.65±0.79 µV, −0.62±0.37 µV, and −2.52±0.90 µV for SS, SL, LS, and LL conditions, respectively (illustrated in Figure 10).

### The Correlations between SC of RTs and Neural Activities

The positive linear correlation between SC of cue-related positivity and stimulus-related negativity was significant (Pearson correlation coefficient (r)* = *0.566, *p = *0.035) in the SS condition. The positive linear correlation between SC of RT and cue-related positivity was significant (r* = *0.541, *p = *0.046) in the SL condition. The negative linear correlation between SC of RT and cue-related positivity was significant (r* = *−0.577, *p = *0.031) in the LS condition. The negative linear correlation between SC of RT and stimulus-related negativity was significant (r* = *−0.760, *p = *0.002) in the LL condition.

### sLORETA Results

Cue-related (and stimulus-related) brain activities for switch and repeat trials were compared to examine the areas involved during the switching process in each condition. Switch-related positivity and negativity brain activities are displayed in Table 1 and Table 2. For the cue-related waveforms, the differential activations between switch and repeat trials appeared in dorsolateral prefrontal cortex (DLPFC, including middle frontal gyrus, superior frontal gyrus, inferior frontal gyrus, and precentral gyrus), left temporal lobe, and posterior parietal cortex (PPC, including inferior parietal lobule, superior parietal lobule, and postcentral gyrus) in the SS, SL and LL conditions. For the stimulus-related waveforms, the differential activations between switch and repeat trials appeared in right frontal lobe, bilateral temporal lobe, and left parietal lobe. Switch trials evoked larger activation in DLPFC, PPC, and left temporal cortices in the SS condition for cue or stimulus-related waveforms. For the SL condition, there was no difference between switch and repeat trials in both waveforms. For the LS condition, switch trials evoked larger activation in DLPFC, PPC, and left temporal cortices for the cue-related ERP and smaller activation in left temporal cortex for the stimulus-related waveform. For the LL condition, switch trials evoked larger activation in DLPFC, PPC, and left temporal cortices for the cue-related ERP, and smaller activation in DLPFC and temporal cortices for the stimulus-related waveform.

**Table 1:** Switch-related brain regions in cue-related ERP positivity.

Brain Regions (BA)	T-value	MNI Coordinates		
		X	Y	Z
***SS: switch > repeat (425–475 ms)***				
**Frontal Lobe**				
R Middle Frontal Gyrus(10)	1.15*	45	53	−7
**Parietal Lobe**				
L Inferior Parietal Lobule (40)	1.20*	−64	−42	25
**Temporal Lobe**				
L Supramarginal Gyrus (40)	1.37*	−59	−48	21
***SL: switch > repeat (400–600 ms)***				
**Frontal Lobe**				
R Middle Frontal Gyrus (10)	2.83**	40	53	−3
L Medial Frontal Gyrus(11/25)	2.79**	−5	33	18
L Superior Frontal Gyrus (6/10)	2.76**	−15	59	20
R Inferior Frontal Gyrus (10)	2.89**	45	48	−2
L Orbital Gyrus (11)	2.88**	−5	38	−23
L Precentral Gyrus (4/6)	2.83**	−45	−12	56
L Rectal Gyrus (11)	2.84**	−5	33	−23
**Temporal Lobe**				
L Transverse Temporal Gyrus (41/42)	3.01**	−59	−19	10
L Superior Temporal Gyrus (22/41/42)	3.01**	−59	−24	1
L Middle Temporal Gyrus (21)	2.95**	−59	−29	−3
**Parietal Lobe**				
L Inferior Parietal Lobule (40)	2.98**	−45	−46	53
R Superior Parietal Lobule (7)	2.92**	30	−55	58
L Superior Parietal Lobule (7)	2.79**	−35	−71	45
L Postcentral Gyrus (1/3/5/40/43)	2.88**	−40	−41	62
***LL: switch > repeat (400–600 ms)***				
**Frontal Lobe**				
R Middle Frontal Gyrus (6/10)	1.84**	30	58	2
R Precentral Gyrus (6)	1.73**	59	2	37
**Temporal Lobe**				
L Middle Temporal Gyrus (37)	1.71**	−54	−63	3
**Parietal Lobe**				
L Precuneus (7/19)	1.75**	−25	−76	41
L Superior Parietal Lobule (7)	1.75**	−30	−65	54
L Supramarginal Gyrus (40)	1.75**	−59	−47	30

Brain regions that showed significant differential activation between switch and repeat trilas are displayed.

* *= *corrected *p*<0.05, ** = corrected *p*<0.01, R* = *right, L* = *left.

BA: Brodmann area; MNI: Montreal Neurological Institute coordinates.

**Table 2:** Switch-related brain regions in stimulus-related ERP negativity.

Brain Regions (BA)	T-value	MNI Coordinates		
		X	Y	Z
***SS: switch > repeat (400–500 ms)***				
**Frontal Lobe**				
R Middle Frontal Gyrus(10)	1.15*	45	53	−7
**Parietal Lobe**				
L Inferior Parietal Lobule(40)	1.15*	−64	−42	25
**Temporal Lobe**				
L Supramarginal Gyrus(40)	1.29*	−59	−48	21
***SL: repeat > switch (250–350 ms)***				
**Temporal Lobe**				
L Middle Temporal Gyrus(21)	−3.91**	−59	−29	−3
***LL: repeat > switch (250–350 ms)***				
**Frontal Lobe**				
R Rectal Gyrus (11)	−1.62*	5	52	−24
R Subcallosal Gyrus (11)	−1.61*	10	24	−10
R Superior Frontal Gyrus (11)	−1.59*	5	52	−19
**Temporal Lobe**				
R Superior Temporal Gyrus (22/42)	−1.17*	64	−14	5
R Middle Temporal Gyrus (21/37)	−1.05*	59	−49	−2

Brain regions that showed significant differential activation between switch and repeat trials are displayed.

* = corrected *p*<0.05,

** = corrected *p*<0.01, R* = *right, L = left.

BA: Brodmann area; MNI: Montreal Neurological Institute coordinates.

## Discussion

The main goal of the current study was to investigate the neural mechanisms underlying the switch cost (SC) using ERPs and sLORETA techniques, focusing on processes of task-set reconfiguration and passive dissipation of the previously relevant task-set. We found that cue-related SC was determined mainly by RSI, reflecting the passive dissipation process, while stimulus-related SC was modulated mainly by CSI, reflecting the process of task-set reconfiguration. In addition, sLORETA demonstarted that dorsolateral prefrontal cortex (DLPFC) and posterior parietal cortex (PPC) evoked higher activation in switch trials than in repeat trials. These findings suggest that task-switching neural activities in frontal-parietal areas are modulated by processes of both task-set and task-dissipation.

### Behavioral and Neural Switch Cost

We found longer response times (RT) in switch trials compared to repeat trials, and with short CSI compared to long CSI, replicating findings of earlier studies [9], [13], [35]. Similar effects of switch trials and short CSI were found in ERPs. Cue-related ERP showed higher activities and stimulus-related ERP showed lower activities in switch trials and with short CSI.

Furthermore, the RT showed that long CSI decreased the SC in comparison with short CSI (Figure 4). This finding is in line with previous reports showing that reconfiguring a new task-set plays a critical role when switching between tasks [36]–[39]. Behavioral SC was larger in the short CSI condition, presumably because of the increased difficulty in preparing for the new task-set, which is consistent with the task-set reconfiguration theory. Behavioral SC did not decrease with increasing RSI, implying that 750 ms for RSI is sufficient for a behavioral response. Since behavioral response is an output of task-switching, it does not reveal all the processes involved in task-switching. In order to compensate for this, we have checked neural switch cost computed from ERPs and found that SC of RTs was related to the neural SC. The neural switch cost (Figure 7, 10) demonstrated smaller amplitude of cue-related positivity, and of stimulus-related negativity, for long RSI and CSI conditions, respectively. Based on these results, we propose that task-switching cost has at least two sources. One is the effect of previous task-set, including the interference of a previously relevant task-set to the current task-set and the inhibition of current task-set by the previously task-set supporting the TSI theory. The other is the reconfiguration of a new task-set and the active preparative process, supporting the TSR theory. Following discussions will point out the role of RSI and CSI on the neural SC more specifically within the framework of the TSI theory and the TSI theory.

### The Role of RSI on Neural Switch Cost of Cue-related ERP Positivity

The process of passive dissipation of a previously relevant task-set is determined by the RSI, which is considered as one of the main factors of the task-switching cost. Although we did not observe significant RSI effects on behavior, we found an RSI effect on a neural correlate for cue-related ERP positivity in the parietal area [14]–[15], [20], [22] that was not observed for the stimulus-related neural SC. We found a reduction in parietal activity for repeat trials, which is likely associated with a cue-related “neural switch cost”. Cue-related neural SC was enhanced in the short RSI condition, presumably because of the increased difficulty in overcoming the interference from the previously relevant task-set, which is consistent with the task-set inertia theory (TSI). These finding suggest that overcoming interference from a previous trial may be related to the cue-related neural SC.

Our study provided novel findings of the relationship between cue-related neural SC and behavioral SC, in which a positive liner correlation appeared in the SL condition (short RSI, long CSI), and a negative linear correlation appeared in the LS condition (long RSI, short CSI). These results indicated that when task-dissipation was more difficult than task-set reconfiguration (SL condition), a larger cue-related neural SC increased behavioral SC, while when the task-set reconfiguration was more difficult than that of task-dissipation (LS condition) a larger cue-related neural SC decreased behavioral SC. On the other hand, cue-related neural SC may facilitate behavioral SC in an easier task-set reconfiguration and constrain behavioral SC in a harder task-set reconfiguration.

The brain topographies of the ERP postitivity (Figures 5, 6) showed a parieto-frontal distribution for the short CSI (150 ms) condition and a parieto-occipital distribution for the long CSI (600 ms) condition. This shift of brain activity from posterior to anterior with increased difficulty of the task is in line with previous research [40]–[43]. Topographical Switch-related differences were mainly in the parietal cortex, supporting the findings that parietal cortex is related with executive control functions [44].

### The Role of CSI on Neural Switch Cost of Stimulus-related ERP Negativity

The anticipatory component of the task-set reconfiguration process was determined by the CSI, which is another major factor of the task-switching cost. We observed a neural correlate of CSI effects on SC characterized by a stimulus-related ERP negativity in central brain areas [14]–[15], [20], [22]. We found that an increase in central brain activity for repeat trials was associated with a stimulus-related “neural switch cost”. Stimulus-related neural SC was smaller in the long CSI, indicating that an increase in preparation time for the task-set reconfiguration resulted in the decrease of the SC, supporting the task-set reconfiguration theory (TSR). These finding may imply that the task-set reconfiguration process is a major determinant of the stimulus-related neural SC.

An interesting finding was the negative linear relationship between stimulus-related neural SC and behaviral SC in the LL condition (long RSI, long CSI), implying that when subjects have comparatively enough time for the processes of task-dissipation and task-set, behavioral SC is related to the stimulus-related neural SC directly. A positive linear relationship was found between stimulus-related and cue-related neural SC in SS condition, suggesting an overlap between the processes of task-set and task-dissipation. Thus, the balance between RSI and CSI may be important for cognitive control.

The brain topographies of ERP negativity (Figures 8, 9) showed a frontal distribution in the short CSI (150 ms) condition and a parieto-occipital distribution in the long CSI (600 ms) condition. Topographical switch-related differences were mainly in parieto-frontal cortex for short CSI and parieto-occipital cortex for long CSI, similar to the findings of the cue-related ERP positivity. These results suggest that there is a frontal shift when the task becomes harder, and support the findings that frontal and parietal cortices are modulated by the processes of task switching [45], [46]–[49].

Overall our findings suggest that RSI modulates task-dissipation (TSI) and may be a key determinant of the cue-related neural SC, while CSI modulates task-set reconfiguration (TSR) and seems to be a major determinant of the behavioral and stimulus-related neural SC.

### Switch-related Frontal-parietal Cortices

sLORETA can provide more spatial information and was used to explore the brain areas involved in task switching. We compared cue-related and stimulus-related brain activation in each condition and our findings revealed that both DLPFC and PPC play a major role in the task switching process. Specifically, Middle Frontal Gyrus (MFG, BA 10) and Inferior Parietal Lobule (IPL, BA 40) were engaged for both trial types, but with a higher activation for switch trials, suggesting that the fronto-parietal network is involved in the task switching process [45], [46]–[50]. During switching, the task-set, stimulus, Stimulus-Response (S–R) mappings and representation of a response is updated, and the conflict between different task sets and the representation of S–R mapping are monitored and resolved. Prefrontal cortex and posterior associated regions are both essential in the configuration of a new task-set, and thus it is likely that both the DLPFC and PPC are involved during task switching.

In addition, there were increased activity in ACC, PPC and DLPFC during switch trials. This suggests that the increased conflict during switch trials activated ACC and PPC, so that more cognitive control resources were required to resolve conflict, and an increased activation of DLPFC was required for increased cognitive control [11], [22], [26]–[27], [31]. Our findings also suggested that the involved, distributed network consists of functional separable brain circuits. For example, the increased activation of DLPFC in the SL condition may reflect the fact that more control resources were required due to time pressure, task-set conflict and representation of S–R mappings (Table 1). Interestingly, in stimulus-related ERP negativity there was an increased activity in frontal and parietal cortices for switch trials only in the SS condition (Table 2), implying that short CSI and short RSI resulted in more involvement of fronto-parietal network.

### Conclusion

We used ERPs and sLORETA to explore the RSI and CSI-related changes in the cost of task-switching. Short CSI produced a larger behavioral and stimulus-related neural SC, while short RSI produced a larger cue-related neural SC. These findings suggest that the process of task-set reconfiguration may be related to neural activities after stimulus onset, while the dissipation process may related to neural activities after cue onset. Furthermore, sLORETA showed that switch trials evoked larger activation than repeat trials in dorsolateral prefrontal cortex (DLPFC) and posterior parietal cortex (PPC), suggesting a modulation effect on frontal-parietal network by task switching. Taken together, these results show that neural activities of task-switching were modulated by both RSI and CSI, however, they seem to have a differential role on the sub-processes of task-set reconfiguration and the passive dissipation of a previously relevant task-set.

## Supporting Information

Table S1 **Cue-related parietal amplitudes.** Mean amplitudes (µV) and the corresponding SEM at electrode site Pz for cue-related waveforms across the 4 conditions in repeat and switch trials.
(DOC)

Table S2 **Stimulus-related central amplitudes.** Mean absolute amplitudes (µV) and the corresponding SEM at Cz/Pz sites for the stimulus-related waveforms across the 4 conditions in repeat and switch trials.
(DOC)
