# Supplementary material for: Neural Mechanisms Underlying the Cost of Task Switching: An ERP Study
Source: PLoS One. 2012 Jul 30;7(7):e42233. doi: 10.1371/journal.pone.0042233 (PMC3408496; doi:10.1371/journal.pone.0042233)
Supplement: Table S1 — Cue-related parietal amplitudes. Mean amplitudes (µV) and the corresponding SEM at electrode site Pz for cue-related waveforms across the 4 conditions in repeat and switch trials. (DOC) [file pone.0042233.s001.doc]

**Table S1. Cue-related parietal amplitudes.** Mean amplitudes (µV) and the corresponding SEM at electrode site Pz for cue-related waveforms across the 4 conditions in repeat and switch trials.

| Condition | Short RSI | | Long RSI | |
| --- | --- | --- | --- | --- |
|  | Short CSI | Long CSI | Short CSI | Long CSI |
| Repeat | 2.93 ± 0.36 | 1.21 ± 0.19 | 4.05 ± 0.74 | 1.38 ± 0.42 |
| Switch | 4.14 ± 0.69 | 3.30 ± 0.54 | 4.49 ± 0.80 | 2.49 ± 0.43 |
