# Supplementary material for: Neural Mechanisms Underlying the Cost of Task Switching: An ERP Study
Source: PLoS One. 2012 Jul 30;7(7):e42233. doi: 10.1371/journal.pone.0042233 (PMC3408496; doi:10.1371/journal.pone.0042233)
Supplement: Table S2 — Stimulus-related central amplitudes. Mean absolute amplitudes (µV) and the corresponding SEM at Cz/Pz sites for the stimulus-related waveforms across the 4 conditions in repeat and switch trials. (DOC) [file pone.0042233.s002.doc]

**Table S2. Stimulus-related central amplitudes.** Mean absolute amplitudes (µV) and the corresponding SEM at Cz/Pz sites for the stimulus-related waveforms across the 4 conditions in repeat and switch trials.

| Condition | Short RSI | | Long RSI | |
| --- | --- | --- | --- | --- |
|  | Short CSI | Long CSI | Short CSI | Long CSI |
| Repeat | 2.28 ± 0.57 | 5.82 ± 0.92 | 2.58 ± 0.57 | 5.75 ± 0.87 |
| Switch | 1.85 ± 0.44 | 3.17 ± 0.29 | 1.96 ± 0.35 | 3.23 ± 0.46 |
